# Efficacy and cost effectiveness of telemedicine for improving access to care in the Paris region: study protocols for eight trials

**DOI:** 10.1186/s12913-016-1281-1

**Published:** 2016-02-08

**Authors:** Nathanael Charrier, Kevin Zarca, Isabelle Durand-Zaleski, Christine Calinaud, the ARS Ile de France telemedicine group

**Affiliations:** 1 Faculty of Medicine, University Paris-Est, Créteil, France; 2 ECEVE UMRS 1123, Paris, France; 3 Hôpital Hôtel Dieu, URC Eco Ile-de-France (AP-HP), 1 Place du Parvis Notre Dame, 75004 Paris, France; 4 Department of Public Health, Henri Mondor-Albert Chenevier Hospitals (AP-HP), Créteil, France; 5 ARS Ile de France, Direction de la stratégie, pôle SI santé et innovations, Paris, Franceᅟ

**Keywords:** Telemedicine, Evaluation Studies, Method

## Abstract

**Background:**

With the development of information and communication technologies, telemedicine has been proposed as a way to improve patient management by facilitating access to appropriate diagnosis and treatment. The Paris Ile de France Regional Health Agency is currently funding a comprehensive program of telemedicine experiments. This article describes the protocols for the evaluation of the implementation of telemedicine in the Paris region.

**Methods/design:**

Over 2,500 patients have been included in eight studies addressing the use of telemedicine in the context of specific diseases or settings. Two projects are randomized controlled trials, while the six other projects are based on before-after designs (differences in differences studies). Based on the MAST model and the French national framework, we identified endpoints to assess the impact of telemedicine on five dimensions: clinical effectiveness, cost-effectiveness, security of the application, patient satisfaction and quality of life and perception of professionals.

**Discussion:**

Telemedicine encompasses a wide range of services and stakeholders, and thus study protocols must be tailored to the specific constraints and interests of the users.

**Trial registration:**

NCT02110433 (03/07/2014), NCT02157740 (05/27/2014), NCT02374697 (02/05/2015), NCT02157727 (05/27/2014), NCT02229279 (08/28/2014), NCT02368769 (02/05/2015), NCT02164747 (NCT02164747), NCT02309905 (11/27/2014).

## Background

Improving access to care for either specific populations or specific conditions or diseases is a strategic priority for the Paris Regional Health Agency (*Agence Régionale de Santé Ile-de-France*), which has authority over health care policy in the capital region of France, with a population of 12 million with a budget of €30 billion. The region has 419 hospitals and 2,000 long term and social care institutions and a total of 190,000 healthcare professionals. Its missions cover prevention, health care delivery and social care.

Telemedicine [1] has been shown to improve the management of chronic conditions, to improve decision making in acute situations [2], to compensate for local scarcity of health care resources [3] and to provide access to rare expertise [4–6]. In France, it was first legally defined in the 2009 Hospital, Patients, Health and Territories Act and the 2010 Social Security Finance Act. Telemedecine involves the remote exchange of data between a patient and health care professionals or between professionals as part of a patient’s diagnosis and health care management and is limited by decree^1^ to four types of intervention (Table 1), all of which are under the responsibility of a physician at the remote site.

**Table 1:** Definition of telemedicine by type of provider and participant (Decree No. 2010–1229 Art.R. 6316–1 of 19 October 2010)

Type of telemedicine	Definition	On site	Remote site
Teleconsulting	Remote consultation	Patient	Physician
		Physician(s) (optional)	
		Health professional(s) (optional)	
Tele-expertise	Expert advice	Physician	Physician
Telemonitoring	Clinical, radiological or biological data interpretation	Patient	Physician
		Physician(s) (optional)	
		Health professional(s) (optional)	
Teleassistance	Medical procedure	Physician(s) and/or	Physician
		Health professional(s)	

A five-year plan with a total budget of €14 million was established by the Paris Regional Health Agency to foster the development of telemedicine in two major areas: promotion of the efficient utilization of health services in people with chronic illness and long-term conditions and improvement of access to specialized care in geographically or socially deprived populations. Chronic illnesses targeted for telehealth programs were congestive heart failure, end-stage renal failure, mental disorders and obesity. Populations were selected on the basis of need for specialized medical advice or care and access problems include neonates, nursing home residents and more generally older people with long-term conditions and social care needs, patients with acute stroke, inmates with health needs and surgical patients with suspected tumors.

The deployment of telemedicine interventions followed a competitive bidding process, and projects were selected based upon the priorities described above. The telemedicine program was also designed to create multidisciplinary teams in health and social services and to implement plans to deliver care or expertise more effectively.

National and international templates for the assessment of telemedicine projects are currently available (MAST [7], WSD [8], HAS [9]). All emphasize the need for a comparative design [10] and acknowledge the trade-off between methodological requirements to ensure internal and external validity on the one hand and feasibility, support of stakeholders and available research funds on the other. All templates also underscore the importance of using a multiple endpoint/multiple stakeholder approach. This approach may be represented by a matrix that combines clusters of endpoints with groups of stakeholders. We report here on the protocols of ongoing studies selected by the Paris Regional Health Agency to deal more efficiently with chronic conditions and to reduce social and geographic inequities in access to care, including the methodological issues raised by these studies.

## Methods/design

The research aim for each of the trials is to assess the effectiveness and cost-effectiveness of telemonitoring and teleconsulting in the management of patients with long-term health conditions, and teleconsulting or teleexpertise in the management of patients with social or geographical limitation to care access. The proposed trials are pragmatic, comparative (randomized or before-after) and assess the impact of the interventions in the context of routine delivery of care in the Paris region.

### Populations, conditions and settings

Five categories of patients are included in the telemedicine trials:

1. **Community-based adult patients**: The telemedicine trials concern patients suffering from heart failure or requiring care following knee or hip replacement surgery for osteoarthritis. These patients need precise and continuous management with follow-up visits or care at home.
2. **Institutionalized patients**: These patients are dependent elderly persons living in nursing homes without on-site access to primary and secondary care. Many consultations currently require medical transportation and the involvement of several healthcare professionals.
3. **Inmates:** Prisoner transportation is hazardous and costly, resulting in a significant lack of and the access to health care. The first phase of the telemedicine experiment aims to deliver dermatological expertise, followed by other specialities once feasibility has been demonstrated**.**
4. **Neonates:** Neonatal ICU patients are frail neonates at high risk of developing premature retinopathy. While these patients require expertise to ensure an early diagnosis, access to specialized paediatric ophthalmology is limited.
5. **Surgical patients needing intraoperative frozen section analysis and patients with lesions requiring complex pathological interpretation:** The scarcity of on-site and specialized pathologists to provide second opinions prompted the decision to use whole slide imaging for remote pathological examination.

Over 2,500 patients will be included in the Paris Regional Health Agency telemedicine trials.

Few exclusion criteria were defined for the studies, and patients will not be excluded based on the existence of additional physical co-morbidities.

### Interventions

We will assess interventions that fall into three of the four legal categories of telemedicine in France. Each study site has separate agreements with technology suppliers, which were chosen following tender offers by the Paris Regional Health Agency. No attempt was made to standardize the technology across study sites.

1. **Teleconsulting** for institutionalized and community-based patients.
  Two projects aim to evaluate the impact of teleconsulting for institutionalized patients and one for community-based patients. Videoconference tools, allowing real-time, face-to-face remote consultations, are installed in patient homes, nursing homes and hospitals.
  - Telemedicine and Geriatrics in the Essonne area (TLM-TMG91 Clinical Trials NCT02164747): Prior to the availability of teleconsulting, when a nursing home resident had a serious health problem, the patient was transferred to the reference hospital emergency unit using a medical transportation vehicle with a high probability that the patient would remain for more than a few hours at the hospital. Teleconsulting allows the caregiver in the patient room to contact a physician, general practitioner or geriatrician and provide data via connected medical devices such as EKGs and blood pressure monitors. The objective of this unscheduled teleconsulting is to determine whether the patient needs to be transferred to the emergency unit or may remain at the nursing home, with or without new medications.
  - Teleconsulting and nursing homes (TLM-EVLINE Clinical Trials NCT02157740): Scheduled specialized consultations are performed through a video conference application that allows on-site consultations instead of transporting the patient for a physician visit. Like the geriatrics project in the Essonne area, unscheduled teleconsulting is also used as a means of determining whether transfer to an emergency unit is necessary.
  - Teleconsulting and rehabilitation at home after hip and knee surgical procedures (TLM HAD Clinical Trials NCT02229279): Patients receive a videoconference tool at home allowing the coordinated care physician to perform scheduled remote consultations, to supervise treatment and health condition after orthopedic surgery and to foster the therapeutic education of patients.
2. **Tele-expertise** for neonates, surgical patients and inmates.
  All three tele-expertise projects focus on imaging based on picture archiving and transfer.
  - Tele-imaging to evaluate retinopathy of premature infants (TLM-DITEROP Clinical Trials NCT02157727): Screening for retinopathy of premature infants (ROP) involves highly specialized pediatric ophthalmologists, who are scarce outside of Paris. Before the recent availability of tele-expertise, it was difficult for neonatology units to follow the international recommendations for ROP screening [11]. When a specialist was not present on site, the infant was sent home with an ophtalmologist appointment. Tele-expertise allows fundus examination to be performed by a trained nurse, with pictures uploaded to a secure server and interpreted by specialized pediatric ophthalmologists.
  - Digital slides and image transfer (TLM-Pathology Expertise NCT02374697 and TLM-Pathology Frozen Section NCT02368769): This project is aimed at performing intraoperative frozen section analysis offsite in community hospitals with the goal of obtaining the same diagnostic accuracy as with original glass slide interpretation. The same technology is used to obtain second opinions from specialized pathologists for complex pathological diagnoses using digital slides uploaded to a webserver.
  - Teledermatology for inmates (TLM-Teledermatology Clinical Trials NCT02309905): There is a lack of dermatologists to treat skin lesions in prisons. The waiting time for an appointment often exceeds one month, and the inmate must be transported by the police to a hospital, with both the location and the date of transfer kept secret in order to prevent escapes. Furthermore, the inmate may not available for various reasons at that the time of the appointment. The objective of tele-expertise is to send images of the lesion to an offsite dermatologist, who can then prescribe an appropriate treatment for the inmate.
3. **Telemonitoring** for patients with heart failure
  Heart Failure Educational and Follow up Platform (HELP Clinical Trials NCT02110433): Connected devices for patients suffering from severe heart failure monitor weight, blood pressure and BNP (Brain Natriuretic Peptide, a biomarker of Heart Failure) and also provide an educational platform for patients and relatives. The aim is to reduce the number of adverse events, including all-cause mortality, unplanned heart failure-related hospitalizations, emergency department admissions.

### Study designs

Two studies among the eight used the individual randomized control trial (RCT) design (Table 2). Nevertheless, such designs often are neither straightforward nor ethical to implement, particularly when the professionals in charge of patient inclusion are not familiar with medical research procedures.

**Table 2:** The eight telemedicine studies

Project	Population	Condition	Setting	Procedures	Study design	Primary Outcome Measures	Sample size
TLM-HAD	Patients with total knee replacement for Osteoarthritis or hip replacement for coxarthrosis	After surgery	Community	Teleconsulting	RCT	Mean transport duration of the specialist	154
HELP	Chronic heart failure patients	Chronic heart failure	Community	Telemonitoring	RCT	Composite end point: unplanned hospitalizations for HF/all-cause death/non-programmed emergency department admission for HF	330
TLM-EVLINE	Elderly	Emergencies, psychiatry	Nursing Home	Teleconsulting	Controlled before-after study	Number of transports to emergency services	300
TLM-TMG 91	Elderly	Emergencies	Nursing Home	Teleconsulting	Controlled before-after study	Number of days hospitalized	180
TLM-DITEROP	Premature infants	Screening for Retinopathy	intensive care unit	Tele-expertise	Controlled before-after study	Screening rate at due date	620
TLM-PATHOLOGY FROZEN SECTION	Surgical patients	Anatomic pathology	operating theatre	Tele-expertise	Uncontrolled before-after study	Time to perform the diagnostic test	224
TLM-PATHOLOGY EXPERTISE	Surgical patients	Anatomic pathology	Pathological services	Tele-expertise	Uncontrolled before-after study	Time to perform the diagnostic test	470
TLM-DERMATOLOGY	Inmates	Dermatology	prison	Teleconsulting	Controlled before-after study	Proportion of patients receiving dermatological expertise among those requiring it	100

A more pragmatic approach is the pre-post (before-after) design, where the addition of a control group reinforces the validity of the results (controlled before-after or differences in differences studies) [11]. Four studies used the latter design, as it allows greater flexibility and produces faster results while ensuring sufficient level of evidence for health authorities. Control groups could not be created for two studies, and thus we used before-after designs without control groups.

For studies with a control group, four groups will be identified:

- The intervention group before the implementation of the telemedicine intervention
- The intervention group during telemedecine intervention
- The control group before implementation of the telemedicine intervention
- The control group during the telemedicine intervention

For studies without a control group, two groups will be identified:

- The group before the implementation of the telemedicine intervention
- The group during the telemedecine intervention

Kidholm et al. suggest that an assessment based on MAST should not be started while the intervention is still being developed and needs improvement [7]; for that reason, there will be a gap of several months between the end of the “before” period and the beginning of the “after” period.

### Data sources

Various sources will be used depending on the design of the study, including web-based Case Report Forms and administrative databases, such as the Statutory Health Insurance national reimbursement database, which includes dates and costs of all medical transport, consultations and DRGs for hospital admissions. The objective was to use the medical information systems available in each setting to minimize the data collection burden.

### Outcomes

1. Clinical effectiveness
  Multiple endpoints were defined based on the particular characteristics of each telemedicine project:
o **Impact on clinical parameters,** such as the WOMAC score [12] (TLM_HAD), will be measured.
o **Impact on clinical management,** such as the number of lost to follow-up and time to consultation, diagnosis, prognosis or therapeutic decision.
2. Resource utilization, cost and cost-effectiveness
  Impact on health care services utilization will be measured when appropriate, particularly in the telemonitoring and teleconsulting projects. These endpoints include, for example, the number of hospitalizations, number of visits to an emergency department, number of medical transports and length of stay.
  The cost analysis will be performed primarily from a societal perspective, with valuation of the cost of hospital stays (including readmissions), emergency visits, outpatient visits and medical transport.
  Payment for telemedicine services has not been formally established in France, and thus the operating costs will be valued from the health provider perspective. These costs include the labor costs related to operation of the telemedicine intervention, professional and patient education and training, investment in equipment, the cost of building alterations and the call center, where relevant.
  The Incremental Cost Effectiveness Ratio (ICER) will relate the overall costs to the primary endpoint for each study.
3. Security of the application
  We created a registry of all problems that impede the proper functioning of the telemedicine interventions, such as unacceptable delays in obtaining required information and network or interface issues.
4. Patient Perspective
  We will use the WSD SUTAQ questionnaire for telemonitoring projects [13] during the last month of follow up. We previously translated that questionnaire into French following the MAST methodology: two forward translations from English to French by two different persons speaking French and English fluently, one back translation by another English-speaking native, and then an expert committee to validate the accuracy of the translation.
  We created a registry of all patients refusing to use telemedicine in order to assess acceptability by each target population.
  We will measure patient quality of life using the French version of the EQ-5D questionnaire as well as disease-specific quality of life instruments, where appropriate.
5. Professional experience
  All professionals using one of the telemedicine applications will be requested to complete a questionnaire of their perceptions regarding it. The questionnaire is based on the MAST questionnaire and was translated into French using the MAST methodology. We will score each item using a six-item Lickert scale.

### Statistical analysis

Sample size calculations were made to demonstrate superiority (seven studies) or non inferiority (one study), based on the estimated difference and standard deviation (or on the estimated proportions) of the primary endpoint between the groups (telemedicine versus usual care). We used an alpha risk at 5 % and depending on the number of patients potentially includible, we chose a beta risk at 10 % or 20 %. The software used for calculation was found on Statstodo Website [14].

Descriptive statistics (frequencies, means ± SD) will be carried out by group, and subgroup analyses will be performed when relevant.

For the RCTs, we will assess the effectiveness of telemedicine by using the intention-to-treat principle, with the inclusion of all participants in the statistical analyses regardless of whether they completed the intervention or the follow-up measurements. Missing data will be imputed using regression imputation techniques if the population size and dataset allow it.

In the before-after studies with control groups, we will perform differences-in-differences statistical tests.

For trials in which we anticipate censored data, for example due to the high mortality risk of the specific population, we will use the log-rank test. In addition, a Cox proportional-hazards regression model will be used to limit confounding factors.

Multivariate regression analyses will be used to assess which institutional, clinical and demographic factors predict the effect of telemedicine on outcomes.

Each item in the questionnaires of patient satisfaction and health care professionals’ perceptions will be analysed separately. No global scoring will be computed.

## Discussion

The Paris Regional Health Agency telemedicine studies are designed to evaluate the efficacy and cost-effectiveness of an innovative regional policy in improving patient access to care and outcomes compared to usual care in a large population. The proposed study is to our knowledge the first to explore the expected benefits of three different types of telemedicine designed to address the needs of patients with chronic conditions and patients with limited access to care.

Compared to published assessments of telemedicine technologies ([8, 15]) using a single type of trial design and uniform data collection, our studies may appear fragmented and heterogeneous. However, the unifying factor is that each features a comparative design combined with the attempt of use of all relevant elements in the matrix of endpoints and stakeholders.

One of the major lessons of our trial design exercise is that evaluating telemedicine as a whole is not possible. As Population, Intervention, Comparison, Outcome and Time (The PICOT model [16]) are different in each project, each intervention must be evaluated separately.

Another lesson is that telemedicine may require significant changes within organizations, which may lead to a lack of acceptability and thus require a long time before routine use is achieved. Indeed, the authors of the MAST model point out that evaluation of a telemedicine application should not be carried out until it is mature [7]. This is a major consideration in interpreting results, as a negative result may be due to weak project adherence by the professionals involved.

### Withdrawn designs

At the beginning of the trial design phase, we considered designs other than individual RCT and before-after with control group. For example, one attractive alternative randomized design for public health interventions is the stepped wedge design [17–19], which is particularly useful when the intervention is thought to do more good than harm and when it is impossible to implement the intervention in half of all clusters simultaneously because of practical, logistical or financial reasons. In a stepped wedge design, an intervention is rolled-out sequentially to the trial participants (either individuals or clusters of individuals) over a number of time periods. The order in which the individuals or clusters receive the intervention is determined at random and, by the end of the random allocation, all individuals or groups will have received the intervention. We were unable, however, to randomize the sequence of centers which had been previously decided by the funder based on the equipment of facilities and the requirements of the industrial partners.

Interrupted time series designs constitute a more pragmatic approach to evaluating an innovation and are particularly relevant when the outcome is obtained from routinely collected data [11] after implementation of telemedicine, but also before, as statistical analyses rely on the detection of a trend break. As the interventions are still at the experimental stage, there is a learning curve, and it is suggested that the users should be familiar with the innovation before evaluating it. In practice, taking into account the data collected earlier than 3–12 months following implementation could provide false negative results. In our studies, use of this design was not possible as no data were routinely collected before implementation.

### Advantages and disadvantages of the proposed designs

Based on the results of the trials, the Paris Regional Health Agency may propose widespread adoption. The primary objective of this broad assessment program is to document the potential benefits of telemedicine in very different settings and across a wide range of end-users. Another objective is to introduce the concept of evidence based medicine in care settings in which its use has previously been limited. Preliminary work on protocols underscored the need for strong governance, staff training and system redesign, including task shifting and cutting across boundaries and financial incentives.

### Perspectives

Other telemedicine projects are pending and will be analyzed using the same framework: telemonitoring following bariatric surgery, tele-expertise for neonate radiology, teleconsulting for disabled children or individuals with pervasive development disorders.

With the exception of the project “Teleconsulting and rehabilitation at home after hip and knee surgical procedures”, all projects described in the paper began including patients as of December 2014. The first results are expected by the end of 2015.

The Paris Regional Health Agency telemedicine studies are still at an experimental phase, and future projects will be focused on identifying which subgroups of patients may benefit most from telemedicine.

### Ethics approval

An ethical review was not considered necessary for French authorities for most studies for various reasons: 1) TLM-TMG91 and TLM-EVLINE because the data source was extracted from the population-wide health database with only anonymous data, 2) TLM-Pathology Expertise, TLM-Pathology Frozen section and TLM-Teledermatology because the endpoint were the professional procedures and not the clinical outcome.

The ethics approval was given for TLM-HAD by the “Comité de Protection des Personnes Ile de France IV”, number 2013/45.

The ethics approval was given for TLM-HELP by the “Comité de Protection des Personnes Ile de France III”, number Am6714-5-3101.

The ethics approval was given for TLM-DITEROP by the “Comité Consultatif sur le Traitement de l’Information en matière de Recherche dans le domaine de la Santé”, number 15.162.

## Abbreviations

HAS: Haute Autorité de Santé
MAST: Model for ASsessment of Telemedicine
RCT: Randomized controlled trial
TLM: Telemedicine
WSD: Whole System Demonstrator

## Footnotes

1 No. 2010–1229 Art.R. 6316–1 of 19 October 2010.
